# Discrimination of cirrhotic nodules, dysplastic lesions and hepatocellular carcinoma by their vibrational signature

**DOI:** 10.1186/s12967-016-0763-6

**Published:** 2016-01-12

**Authors:** Chengyuan Peng, Slávka Kaščáková, Franck Chiappini, Natalia Olaya, Christophe Sandt, Ibraheem Yousef, Didier Samuel, Paul Dumas, Catherine Guettier, François Le Naour

**Affiliations:** Inserm, Unité 1193, 94800 Villejuif, France; Univ Paris-Sud, UMR-S1193, 94800 Villejuif, France; Instituto Nacional de Cancerologia, Bogota, Colombia; SOLEIL Synchrotron, 91192 Gif sur Yvette, France; Centre Hépato-Biliaire, AP-HP Hôpital Paul Brousse, 94800 Villejuif, France; Service d’Anatomopathologie, AP-HP Hôpital Bicêtre, 94275 Le Kremlin-Bicêtre, France

**Keywords:** Diagnosis, Cirrhosis, Dysplastic nodules, Hepatocellular carcinoma, Infrared microspectroscopy, Synchrotron

## Abstract

**Background:**

Hepatocarcinogenesis is a multistep process characterized in patients with chronic liver diseases by a spectrum of hepatic nodules that mark the progression from regenerative nodules to dysplastic lesions followed by hepatocellular carcinoma (HCC). The differential diagnosis between precancerous dysplastic nodules and early HCC still represents a challenge for both radiologists and pathologists. We addressed the potential of Fourier transform-infrared (FTIR) microspectroscopy for grading cirrhotic nodules on frozen tissue sections.

**Methods:**

The study was focused on 39 surgical specimens including normal livers (n = 11), dysplastic nodules (n = 6), early HCC (n = 1), progressed HCC on alcoholic cirrhosis (n = 10) or hepatitis C virus cirrhosis (n = 11). The use of the bright infrared source emitted by the synchrotron radiation allowed investigating the biochemical composition at the cellular level. Chemical mapping on whole tissue sections was further performed using a FTIR microscope equipped with a laboratory-based infrared source. The variance was addressed by principal component analysis.

**Results:**

Profound alterations of the biochemical composition of the pathological liver were demonstrated by FTIR microspectroscopy. Indeed, dramatic changes were observed in lipids, proteins and sugars highlighting the metabolic reprogramming in carcinogenesis. Quantifiable spectral markers were characterized by calculating ratios of areas under specific bands along the infrared spectrum. These markers allowed the discrimination of cirrhotic nodules, dysplastic lesions and HCC. Finally, the spectral markers can be measured using a laboratory FTIR microscope that may be easily implemented at the hospital.

**Conclusion:**

Metabolic reprogramming in liver carcinogenesis can constitute a signature easily detectable using FTIR microspectroscopy for the diagnosis of precancerous and cancerous lesions.

**Electronic supplementary material:**

The online version of this article (doi:10.1186/s12967-016-0763-6) contains supplementary material, which is available to authorized users.

## Background

Hepatocellular carcinoma (HCC) is the sixth most common neoplasm and the second cause of cancer death worldwide [1, 2]. In Western countries, HCC develops in most cases within an established background of cirrhosis related to various etiologies including hepatitis virus infection, high alcohol intake or metabolic diseases. The systematic surveillance of cirrhotic patients by ultrasound aims to the identification of HCC at a very early stage (<2 cm) with the highest probability of long-term cure. It may also lead to the detection of hepatocellular nodules of uncertain diagnosis. Proper definition of these nodules as pre-neoplastic lesions or early HCC has critical implications. Dysplastic lesions should be only followed by regular imaging and only HCC diagnosis enables the application of potentially curative procedures—albeit expensive—such as resection, transplantation and percutaneous ablation. Thus, there is an urgent need to identify better tools to characterize these lesions.

Hepatocarcinogenesis is a multistep process that is characterized in most cirrhotic livers by the progression from hyperplastic regenerative nodules to low grade dysplastic nodules (LGDN), high grade dysplastic nodules (HGDN) and finally small HCC (less than 2 cm) which corresponds either to vaguely nodular well differentiated HCC (WDHCC) so called early HCC or to distinctly nodular moderately differentiated hepatocellular carcinomas [3–9]. The differential diagnosis between high-grade dysplastic nodules (HGDN) and WDHCC still represents a challenge even for experienced pathologists, especially on biopsy specimens as the only discriminative feature is the presence of focal stromal invasion [5].

Promising results have been reported on gene signatures allowing molecular demarcations between low-grade dysplastic nodules, high-grade dysplastic nodules, and early HCC in both Asian and Western patients [10, 11]. However, the clinical use of gene signatures may face some limitations (too high level of technicity, time-consuming and expensive) and thus are not employed in routine at the hospital. The morphological criteria which define early HCC such as cell density, thickness of liver cell plates, presence of pseudoglands, loss of reticulin, CD34 expression, small cell dysplasia and stromal invasion [3, 6] are also present in high grade dysplastic nodules without clearcut frontier between both lesions. As a consequence, the discrepancies for the early HCC diagnosis are frequent even between expert pathologists [12]. Additional immunohistochemical markers as glypican-3 (GPC3), heat-shock protein 70 (HSP70) and glutamine synthetase (GS) were reported to better characterize early HCC [13, 14]. However a recent prospective study has shown that the use of the antibody panel (GPC3, GS and HSP70) only slightly increases the diagnosis accuracy in an expert setting. Indeed, the sensitivity and specificity for early HCC diagnosis were not highly accurate for each marker (GPC3: 57.5 and 95 %; HSP70: 57.5 and 85 %; GS: 50 and 90 %) as well as in combination (GPC3 + HSP70: 40 and 100 %; GPC3 + GS: 35 and 100 %; HSP70 + GS: 35 and 100 %; GPC3 + HSP70 + GS: 25 and 100 %) [15]. Thus, specific biomarkers of early stages of HCC are still lacking [15].

Infrared spectroscopy is based on the determination of absorption of infrared light due to resonance with vibrational motions of functional molecular groups. Biological tissue is essentially made up of lipids, proteins and carbohydrates, all of which have specific absorption bands in the infrared frequency domain. Thus, infrared spectroscopy is a very valuable tool for biochemical investigations. Fourier Transform Infrared (FTIR) microspectroscopy combines IR spectroscopy and microscopy for determining the chemical composition in small sample area. The increase of spectral quality and spatial resolution was achieved using a bright infrared source, as that produced by synchrotron radiation, thus allowing analysis at the cellular and sub-cellular levels [16–18]. FTIR microspectroscopy has been used to investigate subtle changes associated with various biological processes and discriminate between malignant and non-malignant cells in several different tissues, such as cervix, breast, colon and prostate. This method was capable of identifying a valid biochemical difference between healthy tissues and more or less aggressive tumors [19–25].

The development of cirrhosis and HCC from chronic liver diseases induces a series of changes in the chemical composition of cells and tissues. We have shown previously the capability of FTIR microspectroscopy to investigate the chemical composition of liver lesions such as steatosis or cirrhosis [26–29]. The diagnostic potential of FTIR microspectroscopy has been recently highlighted by the quantitative assessment of liver steatosis on tissue sections [30]. In this report, we demonstrated that FTIR microspectroscopy exhibits the potentiality to detect the chemical changes occurring in hepatocarcinogenesis and to discriminate benign cirrhotic nodules, premalignant (dysplastic) and malignant (cancerous) nodules in cirrhotic liver.

## Methods

### Samples and tissue sections

Tissue samples from 11 surgical liver specimens of normal liver obtained from patients with benign liver tumor or from patients with metastases of colorectal cancer, 21 native livers of patients with hepatocellular carcinoma developed on alcoholic (n = 10) or hepatitis C virus (HCV) (n = 11) cirrhosis and 7 additional native livers of patients with hepatitis B virus (HBV) (n = 1), HCV (n = 3) or alcoholic (n = 3) cirrhosis and hepatocellular nodules initially diagnosed as dysplastic nodules (Table 1) were obtained from the Centre de Ressources Biologiques (CRB) Paris-Sud (AFNOR#2011/39938, Paris-Sud University, France). Three among the seven additional native livers also disclosed a progressed HCC in addition with the dysplastic nodule. For cirrhotic patients, investigations were performed on non-tumor cirrhotic liver and on every type of hepatocellular nodule. The institutional review board approved the study and written informed consent was obtained from all patients. Access to this material was in agreement with French ethical laws.

**Table 1:** History of patients and origin of samples

Patient	Gender	Age (year)	Tissue samples	Associated diagnosis	SR-FTIR	Internal source
1	F	31	Normal liver	FNH	+	+
2	F	26	Normal liver	FNH	+	+
3	F	25	Normal liver	FNH	+	+
4	M	59	Normal liver	Metastasis (GC)	+	+
5	F	37	Normal liver	FNH	+	−
6	M	50	Normal liver	Metastasis (CRC)	−	+
7	F	26	Normal liver	FNH	−	+
8	F	39	Normal liver	Hepatic adenoma	−	+
9	F	54	Normal liver	FNH	−	+
10	F	30	Normal liver	FNH	−	+
11	M	61	Normal liver	Metastasis (CRC)	−	+
12	M	62	Cirrhosis/HCC	Alcohol	+	+
13	M	60	Cirrhosis/HCC	Alcohol	+	+
14	M	58	Cirrhosis/HCC	Alcohol	+	+
15	M	49	Cirrhosis/HCC	Alcohol	−	+
16	M	84	Cirrhosis/HCC	Alcohol	−	+
17	M	57	Cirrhosis/HCC	Alcohol	−	+
18	M	60	Cirrhosis/HCC	Alcohol	−	+
19	M	61	Cirrhosis/HCC	Alcohol	−	+
20	M	61	Cirrhosis/HCC	Alcohol	−	+
21	M	67	Cirrhosis/HCC	Alcohol	−	+
22	M	49	Cirrhosis/HCC	HCV	−	+
23	F	80	Cirrhosis/HCC	HCV	−	+
24	F	66	Cirrhosis/HCC	HCV	−	+
25	F	77	Cirrhosis/HCC	HCV	−	+
26	F	56	Cirrhosis/HCC	HCV	−	+
27	F	59	Cirrhosis/HCC	HCV	−	+
28	M	68	Cirrhosis/HCC	HCV	−	+
29	M	61	Cirrhosis/HCC	HCV	−	+
30	M	71	Cirrhosis/HCC	HCV	−	+
31	M	65	Cirrhosis/HCC	HCV	−	+
32	F	66	Cirrhosis/HCC	HCV	−	+
33	F	58	Cirrhosis/HGDN with nodules of HCC	HCV	−	+
34	M	59	Cirrhosis/HGDN	Alcohol	−	+
35	M	48	Cirrhosis/Early HCC	HCV	−	+
36	M	55	Cirrhosis/HGDN	HBV	−	+
37	M	59	Cirrhosis/HGDN/HCC	Alcohol	+	+
38	M	66	Cirrhosis/LGDN/HCC	Alcohol	−	+
39	M	53	Cirrhosis/HGDN/HCC	HCV	−	+

*FNH* focal nodular hyperplasia, *CRC* colorectal cancer, *GC* gallbladder cancer, *HBV* hepatitis B virus, *HCV* hepatitis C virus, *HCC* progressed HCC, *LGDN* low grade dysplastic nodule, *HGDN* high grade dysplastic nodule

Samples were all obtained at the time of surgery. Samples from normal liver, non-tumor cirrhotic parenchyma and hepatocellular nodules in cirrhotic livers were split into two pieces: one immediately snap frozen in liquid nitrogen and stored at −80 °C until use; the second was fixed in formalin and embedded in paraffin wax for pathological assessment by expert pathologist.

Serial sections were cut from frozen specimens with 5 µm thickness at −20 °C with a CM3050-S cryostat (Leica Microsystèmes SAS, France) and alternately deposited on glass slide for histological control or on a gold coated sample holder MirrIR (Tientascience, Indianapolis, IN) for FTIR microspectroscopy [26–31]. Sections for microspectroscopy were placed in a desiccator loaded with silica gel for at least 4 days. They were dried in the dark environment at room temperature. After drying, samples were stored in a N_2_ atmosphere until used. Sections for histology were stained with hematoxylin, eosin and safran (HES).

Dysplastic nodules were systematically reviewed by two pathologists (Dr. Natalia Olaya and Pr. Catherine Guettier) on paraffin sections stained with HES and with Gordon-Sweet silver impregnation. Additional immunostaining was performed on deparaffinized sections after antigenic restoration by heating in citrate buffer pH6 with antibodies directed against CD34 (clone QBEnd-10, Dako), glypican-3 (GPC-3), (clone 1G12, Zytomed), glutamine synthetase (GS) (clone 6, Biosciences) and HSP70 (clone 2A4, Abcam) using a Bond Max automate based on a labeled streptavidin–biotin (LSAB) method. Nodules were diagnosed as low-grade dysplastic nodules, high-grade dysplastic nodules or well differentiated hepatocellular carcinoma so called early carcinoma according to the International Consensus Group for International neoplasia criteria [5] (Table 2).

**Table 2:** Histology of dysplastic lesions

Patient	Nodule size (cm)	Associated HCC	Portal tracts 0/1	Unpaired arteries 0/1/2	Cell density	Trabeculae thickness	Pseudo-glands 0/1	Steatosis (%)	Large cell dysplasia	Small cell dysplasia	Stromal invasion 0/1	Nodule in the nodule	CD34 (%)	Glypican3 (%)	Glutamine synthetase (%)	HSP70 (%)	Pathological diagnosis
33	2.4	Yes	1	2	X4	>3	1	5	0	1	1	HCC nodules	80	40	25	15	HGDN with HCC nodules
34	2.4	No	1	1	X1.5	2	0	0	0	1	0	0	70	10	70	0	HGDN
35	2.7	Yes	1	0/1	X2	2–3	1	10	0	1	1	0	10	0	15	5	early HCC
36	3.4	No	1	1	X2	2–3	1	25	0	1	0	0	10	10	0 (zonal)	0	HGDN
37	2	Yes	0	0/1	X1.5	2–3	0	0	0	1	0	0	0	5	0 (zonal)	0	HGDN
38	1	Yes	1	0	X1.5	2	0	0	0	0	0	0	40	0	0 (zonal)	0	LGDN
39	1.1	Yes	rares	1	X2	focally > 3	1	0	0	1	0	0	30	0	0 (zonal)	0	HGDN

*HCC* hepatocellular carcinoma, *LGDN* low grade dysplastic nodule, *HGDN* high grade dysplastic nodule

### FTIR microspectroscopy

FTIR microspectroscopy was performed in transflection mode. In transflection mode, the sample is placed on an inexpensive IR-reflecting surface and measurements are generated by a beam passing through the sample and reflecting back from the surface (i.e. the reflective surface) through the sample [31].

Synchrotron infrared microspectroscopy was performed at the SMIS beamline at the SOLEIL Synchrotron Radiation Facility (Saint-Aubin, France) operating at 2.75 GeV with a current of 400 mA delivered in Top-Up mode. Infrared photons were emitted by the electrons deflected from a bending magnet in the storage ring. The infrared photon source was coupled to a Thermo Fischer NEXUS FTIR spectrometer Nicolet 5700. Attached to the spectrometer was a microscope CONTINUUM XL (Thermo Scientific, CA, USA). The detector of the infrared microscope was a liquid nitrogen cooled mercury cadmium telluride (MCT-A) detector (of 50 µm in size). The microscope operated in confocal mode, using a 32× infinity corrected Schwarzschild objective (NA = 0.65) and a matching 32× condenser. All spectra were obtained using a double path single masking aperture (confocal arrangement) size set to 10 µm × 10 µm. The spectra were collected in the 4000–800 cm^−1^ mid-infrared range at a resolution of 4 or 8 cm^−1^ with 16–50 co-added scans. Each spectrum was recorded in approximately 10–25 s. Details of the experimental procedure have been already described [26–29]. Infrared microspectroscopy was also performed on iN10MX microscope equipped with an internal thermal source (Thermo Scientific) for recording large maps. All spectra were collected by ultra-fast mode using 50 µm × 50 µm aperture. The spectra were collected in the 4000–800 cm^−1^ mid-infrared range at a resolution of 16 cm^−1^ with 1 spectrum per pixel [28]. Infrared spectra were acquired at least on two maps of 500 pixels for each tissue section. IR spectra were collected only on the hepatocellular parenchyma avoiding the portal tracts in normal liver and the fibrous septa in cirrhotic liver.

### Data preprocessing and statistical analysis

The infrared images were incorporated into OMNIC version 7.2 (Thermo Scientific) software in order to extract the spectral data that were deemed suitable for further analysis. The spectra first underwent a quality test whereby spectra that had maximum absorbance values outside the range 0.4–1.0 were rejected. This was to ensure that measurements with a good signal to noise ratio and spectral signal within a Beer-Lambert law range were used in further steps. Similarly, the spectra, which showed the presence of Mie scattering, were rejected. After the quality test, for each patient we obtained at least 200 spectra measured by synchrotron source and 800 spectra when the globar source was used. The spectra, which passed the quality test, underwent further preprocessing in The Unscrambler X version 10.1 (CAMO Process AS, Norway) software package. They were converted to their first derivatives with a Savitsky-Golay algorithm, followed by range normalization. The spectral data were mean-centered and then statistically analyzed by principal component analysis (PCA). The spectral intervals considered to discriminate IR spectra were selected by unsupervised criteria. The mean spectra were baseline corrected and normalized using the unit vector normalization in The Unsrambler X. The integrals reflecting peak areas were considered to calculate the ratio values. The *t* test was calculated using the Microcal Origin, version 8.0 program (Microcal Software Inc., Northampton, MA).

## Results

### Discriminating cirrhosis and hepatocellular carcinoma by synchrotron radiation infrared microspectroscopy

**Fig. 1:**
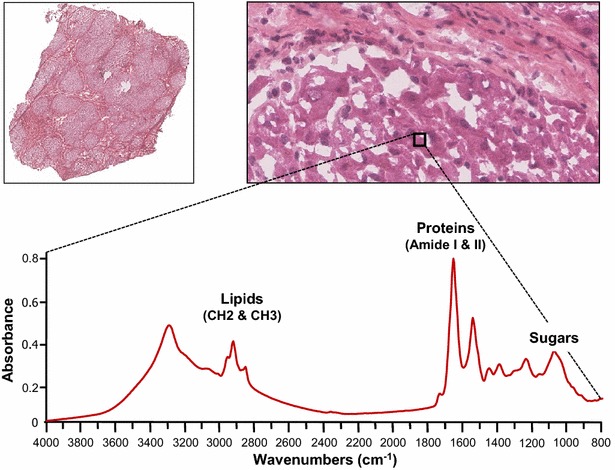
SR-FTIR analysis on cirrhotic liver. Tissue sections of 5 µm thickness were performed from cirrhosis and stained with HES (hematoxylin, eosin and safran). Cirrhosis exhibiting nodules surrounded by fibrosis are shown at ×40 magnification (*upper left*). Synchrotron-FTIR was performed on tissue section of cirrhosis at the resolution 10 µm × 10 µm (*black square on upper right*). The infrared spectrum is shown

Cirrhosis and hepatocellular carcinoma (HCC) were investigated by Fourier transform infrared microspectroscopy using synchrotron radiation (SR-FTIR). The high resolution obtained using synchrotron radiation allows addressing the chemical composition at the cellular level (Fig. 1) and thus leads to investigate in depth the variance in tissues. Spectra were acquired on frozen tissue sections from normal livers, cirrhotic livers and their corresponding HCC (Table 1), and the variance was addressed by principal component analysis (PCA). This unsupervised multivariate statistical method decomposes the data into a weighted sum of uncorrelated principal components (PCs). In the data set coordinate system, the first PC becomes the first axis of a new coordinate system and explains the highest variance of the data set. The second PC becomes the second axis, orthogonal to the first one, and explains the next highest variance of the data set, and so on [32, 33].

**Fig. 2:**
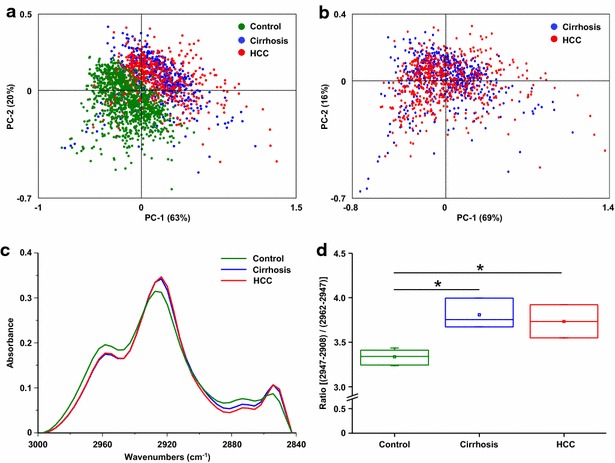
Spectroscopic and statistical analysis on lipids. Spectra were acquired using SR-FTIR on normal livers as controls (n = 5), cirrhosis and their corresponding HCC (n = 3). All spectra were baseline corrected within the 3000–2840 cm^−1^ region. Principal component analysis (PCA) was performed on spectra from **a** normal livers, cirrhosis and HCC or **b** only from cirrhosis and HCC. The score plot based on PC1 and PC2 is shown where each point represents one spectrum. **c** The average spectra corresponding to normal liver, cirrhosis and HCC tissues were extracted. **d** The ratio of two main peak areas corresponding to C–H asymmetric stretch of CH_2_/CH_3_ within each group of tissue was calculated and plotted as *boxplot*. Unpaired Student *t* test was applied between control and cirrhosis/HCC. *: unpaired *t* test, p < 0.05

The study was first focused on lipids (2800–3000 cm^−1^) in IR spectra. Discrimination between the IR spectra of normal liver (control group) and pathological tissues (cirrhosis and HCC) was observed (Fig. 2a, b). The average spectrum corresponding to the normal tissue, cirrhosis or HCC lead to observe a lower intensity of the peaks corresponding to CH_3_ motions (2872 and 2957 cm^−1^) in the pathological tissues whereas the peaks attributed to CH_2_ vibrations (2851 and 2920 cm^−1^) were higher (Fig. 2c; Additional file 1: Table S1). The value of the ratio CH_2_/CH_3_ was calculated and plotted demonstrating the significant increase of CH_2_ relative intensity in cirrhosis and HCC as compared to the healthy tissue (Fig. 2d). These results suggested that the development of cirrhosis is accompanied by important changes of lipids with longer carbon chains.

**Fig. 3:**
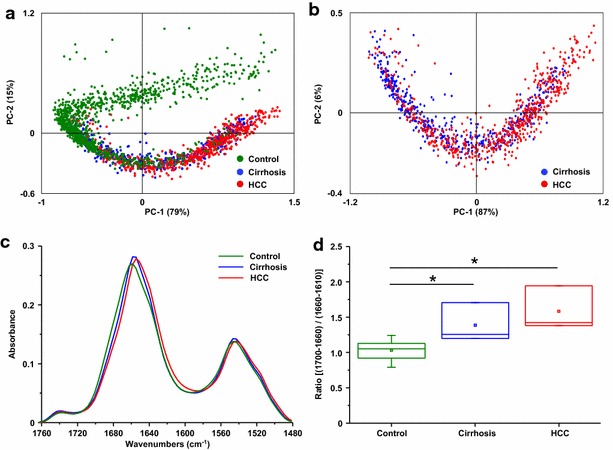
Spectroscopic and statistical analysis on proteins. Spectra were acquired using SR-FTIR on normal livers as controls (n = 5), cirrhosis and their corresponding HCC (n = 3). All spectra were baseline corrected within the 1760–1480 cm^−1^ region. Principal component analysis (PCA) was performed on spectra from **a** normal livers, cirrhosis and HCC or **b** only from cirrhosis and HCC. The score plot based on PC1 and PC2 is shown where each point represents one spectrum. **c** The average spectra corresponding to normal liver, cirrhosis and HCC tissues were extracted. **d** The ratio of two areas of Amide I band was calculated and plotted as *boxplot* highlighting the Amide I peak position shifts within each group. Unpaired Student *t* test was applied between control and cirrhosis/HCC. *: unpaired *t* test, p < 0.05

The IR frequency domain 1475–1710 cm^−1^ attributed to the content in proteins was also investigated. PCA discriminated the vibrational signatures of normal and pathological liver (Fig. 3a, b). The average spectrum of each group revealed a gradual downward frequency shift of the bands Amide I and Amide II between normal liver, cirrhosis and HCC (Fig. 3c). This shift was quantified by displaying the area ratio (1700–1660/1660–1610) demonstrating a significant variation between normal liver and cirrhosis which was accentuated in HCC (Fig. 3d). These observations suggest that changes occur in the proteome in the course of cirrhosis and hepatocarcinogenesis.

**Fig. 4:**
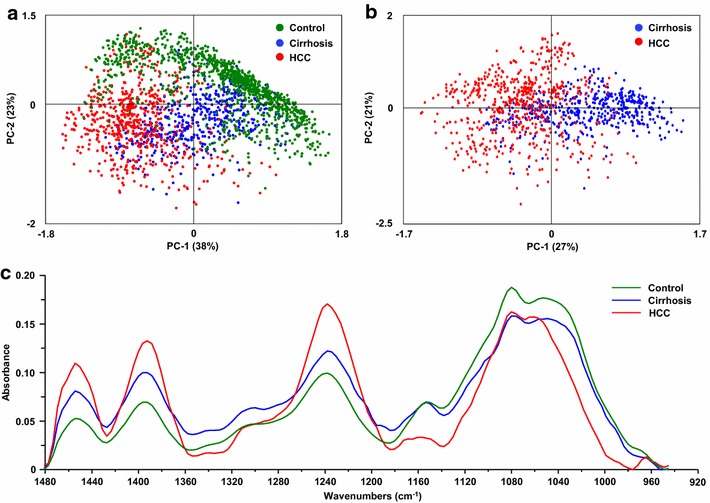
Spectroscopic and statistical analysis on the fingerprinting region. Spectra were acquired using SR-FTIR on normal livers as controls (n = 5), cirrhosis and their HCC (n = 3). All spectra were baseline corrected within the 1480–950 cm^−1^ region. Principal component analysis (PCA) was performed on spectra from **a** normal liver, cirrhosis and HCC or **b** only from cirrhosis and HCC. The score plot based on PC1 and PC2 is shown where each point represents one spectrum. **c** The average spectra corresponding to normal liver, cirrhosis and HCC tissues were extracted and superimposed

Investigations were also performed on the frequency domain 950–1480 cm^−1^. This domain is characterized by bands overlapping lipids, proteins, nucleic acids and carbohydrates (Additional file 1: Table S1). PCA on normal liver, cirrhosis or HCC displayed distinct groups which also discriminated normal and pathological liver tissues (Fig. 4a). Interestingly, cirrhosis and HCC were identified by PCA on infrared spectra. Indeed, 27 % of the variance was observed on the first component (PC1) allowing a valuable separation of the two pathological statuses (Fig. 4b). According with these observations, the average spectra of the frequency domain 950–1480 cm^−1^ exhibited major variations in the intensity and frequency of several peaks (Fig. 4c). These results manifested the marked chemical changes occurring during carcinogenesis and supported the potential of infrared spectroscopy for discriminating cirrhotic and cancerous hepatocytes.

### Discriminating dysplastic nodules from cirrhosis and hepatocellular carcinoma by synchrotron radiation infrared microspectroscopy

**Fig. 5:**
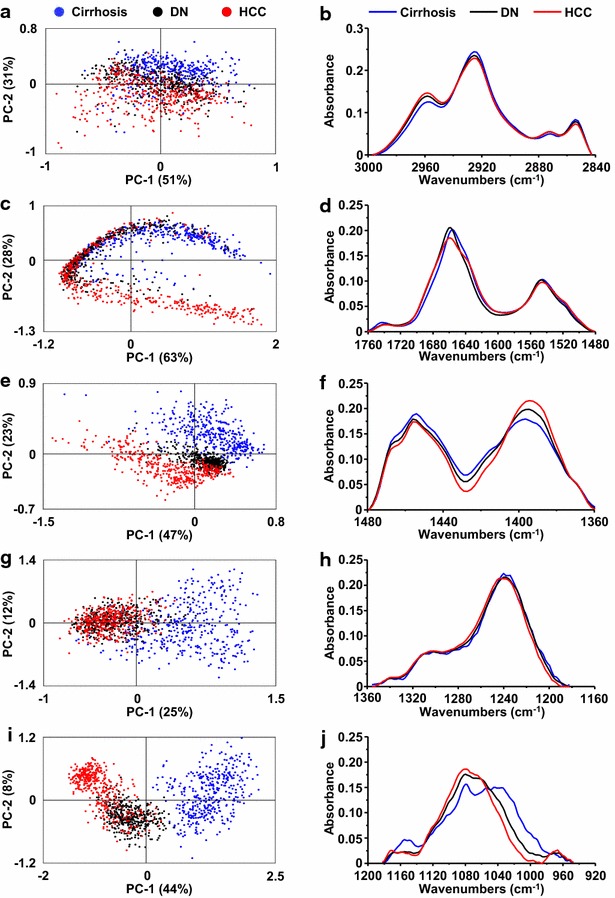
Spectroscopic and statistical analysis on a patient exhibiting hyperplastic nodules, dysplastic lesions and hepatocellular carcinoma. Spectra were acquired using SR-FTIR on frozen tissue sections from the patient #37 and principal component analysis (PCA) was performed on the frequency domains **a** 3000–2840 cm^−1^, **c** 1760–1480 cm^−1^, **e** 1480–1360 cm^−1^, **g** 1360–1180 cm^−1^ and **i** 1180–950 cm^−1^. The average spectra corresponding to cirrhotic, dysplastic (DN) and tumor nodules (HCC) were extracted and superimposed (**b**, **d**, **f**, **h**, **j**)

The possibility to distinguish HCC from cirrhosis by SR-FTIR led to investigate the potential of infrared spectroscopy for discriminating early stages of cancer. Thus, the study was focused on dysplastic lesions, which correspond to precancerous stages. Acquisitions of IR spectra were performed on frozen tissue sections of cirrhotic nodules, high-grade dysplastic nodule and hepatocellular carcinoma from the same patient with HCV cirrhosis (patient #37, Tables 1, 2). Principal component analysis was performed on the frequency domains corresponding to lipids, proteins and sugars (Fig. 5) as described above. On the fingerprinting region 950–1480 cm^−1^, PCA was performed on three distinct frequency domains: 1360–1480, 1180–1360 and 950–1180 cm^−1^. In all frequency domains, a clear discrimination of IR spectra was observed between cirrhosis and HCC originating from the same patient. Interestingly, the spectra acquired on high-grade dysplastic nodule behaved intermediately between cirrhosis and HCC or partially superimposed with those from HCC (Fig. 5). Moreover, the PCA and average spectra obtained on the frequency domains 1360–1480 and 950–1180 cm^−1^ appeared as the most discriminative for cirrhosis, dysplastic nodule and HCC (Fig. 5). These frequency domains correspond respectively to overlapped vibrations from lipids and proteins, and to sugars (Additional file 1: Table S1). Furthermore, the most important variation in the sugars was observed on the bands 950–1050 cm^−1^ attributed to the glycogen content suggesting important changes in such a component in the tumor progression. These results demonstrated that infrared spectroscopy allows grading hepatocellular nodules in cirrhotic liver thus leading to discriminate dysplastic lesions.

### From the synchrotron to a laboratory-based infrared source

**Fig. 6:**
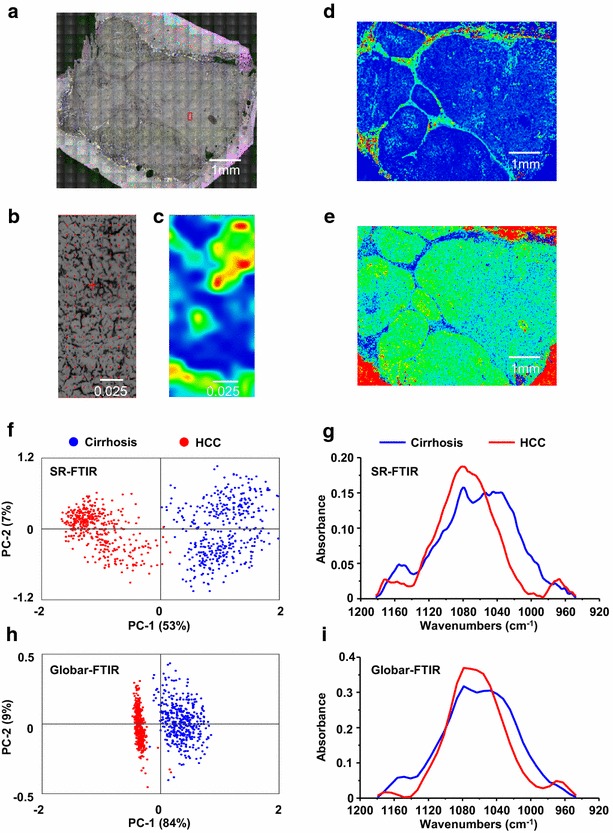
FTIR microspectroscopy on tissue section from cirrhosis and hepatocellular carcinoma. **a** Mosaic optical image on cirrhosis from patient #37. The small area probed with SR-FTIR (*red rectangle*) has been magnified several times (**b**) to investigate chemical composition at high spatial resolution (**c**). **d**, **e** Chemical images obtained mapping whole specimen with globar-FTIR. Contour plots represent the quantitative 2D distribution of collagen (**d**) and lipids (**e**) obtained integrating IR signals within the 1300–1200 cm^−1^ and the 3000–2800 cm^−1^ spectral domains, respectively (*blue* low; *red* high). **f**, **h** PC score plots (each point represents one spectrum) and **g**, **i** the corresponding average spectra in samples probed with SR-FTIR (**f**, **g**) and globar-FTIR (**h**, **i**), respectively

**Fig. 7:**
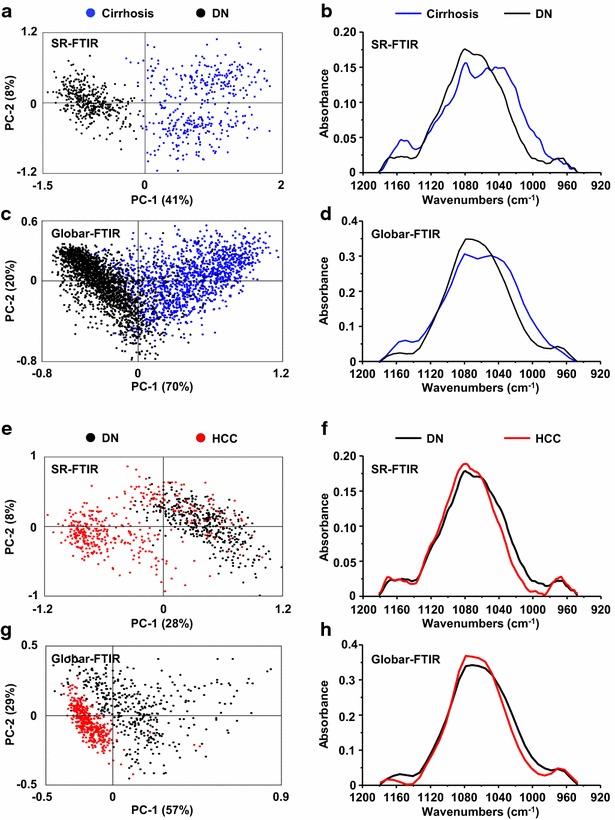
Discrimination between various cirrhotic nodules using synchrotron radiation or a laboratory-based infrared source. Spectra were acquired on frozen tissue sections from the patient #37 using SR-FTIR (**a**, **b**, **e**, **f**) or using a Globar-FTIR microscope (**c**, **d**, **g**, **h**). Principal component analysis (PCA) was performed on the frequency domain 1180–950 cm^−1^. The score plot based on PC1 and PC2 is shown where each point represents one spectrum (**a**, **c**, **e**, **g**) and the average spectra were extracted and superimposed (**b**, **d**, **f**, **h**). *DN* dysplastic nodules, *HCC* hepatocellular carcinoma

The potential of synchrotron radiation infrared microspectroscopy for discriminating dysplastic lesions represents a great interest for the diagnosis of precancerous lesions on patients with cirrhosis. However, the limited access to such a large-scale facility prohibits an extensive use of the technique as a routine diagnostic tool for hospital purpose. We have addressed this issue and used a laboratory-based infrared source on a microscope that can be easily positioned in a clinical environment. Although such an instrument exhibits a poorer lateral resolution as compared to the bright synchrotron radiation, the device allows rapid acquisition of spectra on the whole tissue section leading to a global picture of the composition of the tissue (Fig. 6a–e). The potential of a laboratory-based infrared source was investigated first to discriminate cirrhosis and hepatocellular carcinoma. Acquisitions were performed on the same tissue sections (Table 1) either using synchrotron radiation or using the IR lab-source. Spectroscopic and statistical analysis using PCA were investigated on the frequency domains 1360–1480 and 950–1180 cm^−1^. As expected, a much less variance was observed in the spectra acquired with the laboratory-based IR source. However, the discrimination between cirrhosis and HCC remained possible (Fig. 6f–i; Additional file 2: Figure S1). Studies were further performed on patients exhibiting dysplastic lesions (Tables 1, 2) including the patient #37 that was investigated by both synchrotron radiation and the IR lab-source. Interestingly, the dysplastic nodules were still discriminated from either benign or cancerous lesions (Fig. 7; Additional file 2: Figure S1).

### Characterization of quantifiable spectral markers for diagnosis of benign, dysplastic and malignant lesions

**Fig. 8:**
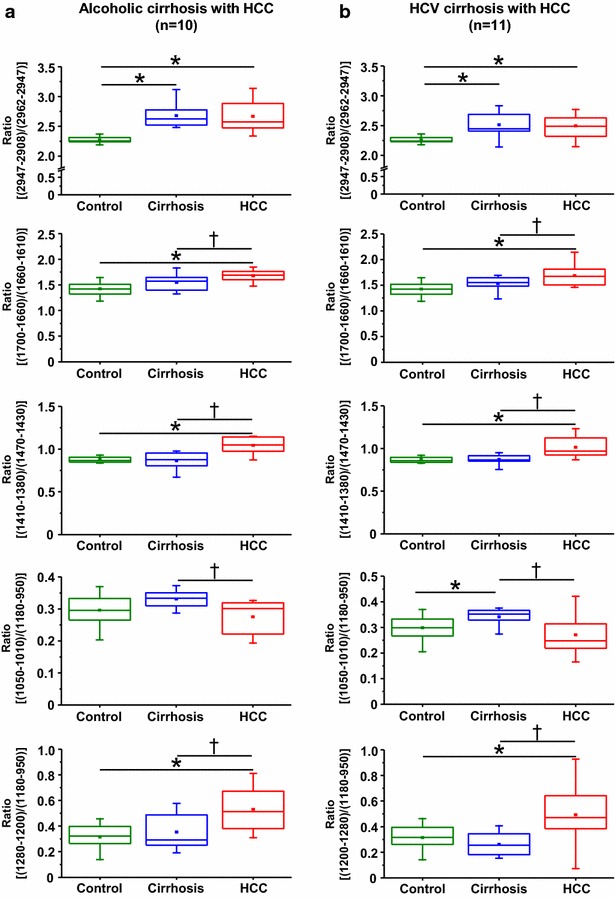
Characterization of band ratios as quantifiable spectral markers. Spectra were acquired on frozen tissue sections using a laboratory microscope FTIR (iN10Mx) equipped with an internal infrared source. Analysis were performed from normal livers as controls (n = 11), **a** alcoholic cirrhosis with their corresponding HCC (n = 10) or **b** HCV cirrhosis and their corresponding HCC (n = 11) (**b**). Ratios of two peaks area were calculated and boxplots are shown. Statistical analysis were performed on all patients with unpaired *t* test (*p < 0.05) or paired *t* test (^†^p < 0.05)

The possibility of grading hepatocellular nodules in cirrhotic livers using laboratory-based infrared microspectroscopy opens new avenue for diagnosis. We sought to define quantitative markers related to the pathological status of the tissue by the calculation of ratio between the areas of two bands in the IR spectrum. This approach exhibited the advantage to provide values independent of the conditions of acquisition (e.g. variations in the thickness of tissue sections). Spectroscopic analysis was undertaken on series of 32 liver surgical specimens including 11 normal livers and 21 cirrhotic livers with HCC (Table 1). Five ratios were investigated in order to take advantage of complete information contained in the IR spectra, which allowed distinguishing normal livers to pathological livers. Moreover, four ratios allowed significant discrimination between cirrhosis and hepatocellular carcinoma. In particular, the three ratios (ratio 1: 1010–1050/950–1180, ratio 2: 1200–1280/950–1180 and ratio 3: 1380–1410/1430–1470) calculated in the fingerprinting region of the IR spectrum led to the most significant separation of cirrhosis and HCC whatever the alcoholic or viral aetiology (Fig. 8).

**Fig. 9:**
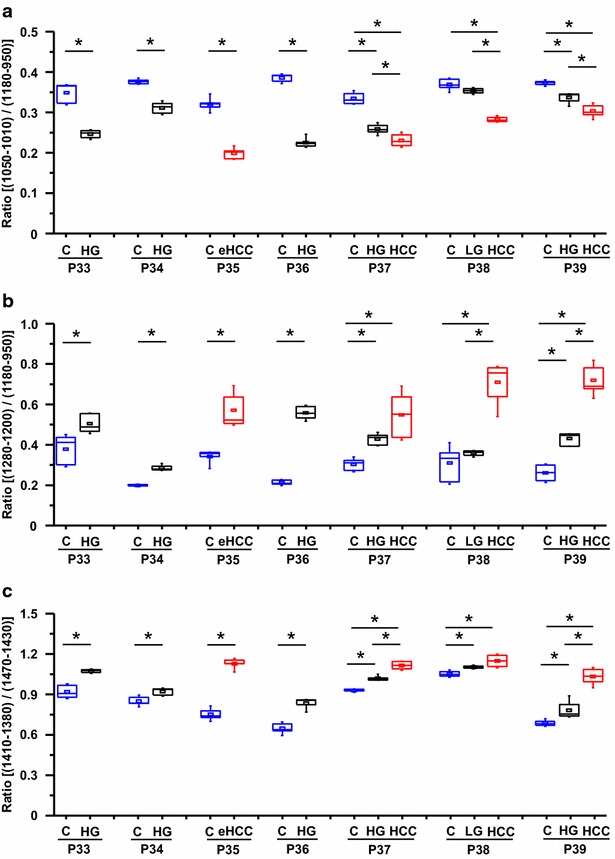
Discrimination between cirrhosis, dysplastic lesions and hepatocellular carcinoma using quantifiable spectral markers. Seven patients exhibiting benign or dysplastic nodules were investigated by FTIR microspectroscopy using a FTIR microscope equipped with an internal infrared source. For each patient, acquisitions were performed on 5 maps with about 200 spectra per map. **a** *Boxplots* of ratio calculated by two areas (1050–1010 cm^−1^/1180–950 cm^−1^), baseline of 1180–950 cm^−1^. **b** *Boxplots* of ratio calculated by two areas (1280–1200 cm^−1^/1180–950 cm^−1^), baseline of 1360–950 cm^−1^. **c** *Boxplots* of ratio calculated by two main peak areas (1410–1380 cm^−1^/1470–1430 cm^−1^), baseline of 1480–1360 cm^−1^. Statistical analysis was performed on each individual patient with paired *t* test (*p < 0.05). *C* cirrhosis, *HG* high grade dysplastic nodule, *LG* low grade dysplastic nodule, *eHCC* early hepatocellular carcinoma, *P* patient

The study was further performed on tissue samples from 7 patients (Table 2) exhibiting low-grade dysplastic nodule (LGDN) (n = 1), high-grade dysplastic nodules (HGDN) (n = 5) and early HCC (n = 1) on cirrhotic livers. Three among these patients disclosed a concomitant HCC. The comparison between benign cirrhotic nodules and high grade dysplastic nodules using infrared microspectroscopy was focused on the three ratios in the fingerprinting region of the IR spectrum. Interestingly, a significant discrimination was observed when comparing regenerative cirrhotic nodules with dysplastic nodules. Furthermore, it was also possible using any of the three ratios to discriminate dysplastic lesions especially HGDN from HCC (Fig. 9).

These results demonstrated the high potential of IR microspectroscopy as a method for discriminating cirrhotic, dysplastic and carcinomatous nodules thus providing reliable assessment of the pathological status of the tissue.

## Discussion

This is the first report investigating the morphological steps of hepatocarcinogenesis in human by using IR microspectroscopy. Profound alterations of the biochemical composition of the pathological liver were highlighted by IR microspectroscopy. Indeed, major changes were observed in lipids, proteins and sugars strengthening the metabolic reprogramming in carcinogenesis [34, 35]. The metabolic anomaly originally described in cancer cells was the up-regulation of glycolysis [36]. The persistent metabolism of glucose to lactate even in aerobic condition has been suggested as an adaptation to hypoxia in cancerous lesions. Thus, subsequent cell populations with up-regulated glycolysis have a powerful growth advantage, which promotes unconstrained proliferation and invasion [37–40]. It has been hypothesized that the altered metabolism specific to cancer cells arose from mitochondrial defects. With this regard, liver carcinogenesis involves a depletion of cellular mitochondrial content that may be related to the repression of the cellular program responsible for proliferation of mitochondria [41]. However, the metabolic signatures of cancer cells are not only passive responses to damaged mitochondria but also result from oncogene-directed metabolic reprogramming required for supporting macromolecular synthesis and anabolic growth. Indeed, the overexpression of the rate-limiting transporter for glucose uptake GLUT1 has been reported in HCC [42] while the gene encoding hexokinase II which catalyzes the first reaction of glycolysis converting glucose to glucose-6-phosphate was shown amplified in hepatoma cells [43]. Metabolic alterations in cancer cells do not concern only glycolysis but impact also in depth the lipid metabolism and the composition of the proteome. De novo lipogenesis in mammalian cells depends on mitochondrial citrate production and fatty acid synthase amplification has been reported in cancer [35]. Several amino acid precursors are derived from the transamination of mitochondrial intermediates. Oxaloacetate can be transaminated to produce aspartate which can serve as precursor for asparagine and alpha-ketoglutarate can be transaminated to produce glutamate, which in turn can be converted to proline, arginine and glutamine [35, 44]. Most cancers depend on these syntheses rather than exogenous supplies. According with all these observations, we have demonstrated that IR microspectroscopy allows revealing lower amount of sugars (i.e. glycogen), changes in proteins and higher amount of lipids reflecting the metabolic changes in glycolysis, protein synthesis and lipogenesis. Thus, IR microspectroscopy provides a global picture of the metabolic reprogramming that is now considered as a hallmark of cancer [45].

Metabolic reprogramming in liver carcinogenesis can constitute a signature easily detectable using IR microspectroscopy for histopathologic diagnosis of precancerous and cancerous lesions. Indeed, IR microspectroscopy represents the only technique allowing quantitative investigations on the global biochemical composition in lipids, proteins and sugars in a single experiment. It exhibits the major advantage to be compatible with tissue sections performed in routine at the hospital without any staining or additional sample preparation. As compared to gene-expression analysis or proteomics, IR microspectroscopy permits in situ studies of heterogeneous tissues at the cellular level thus combining the advantages of conserving the morphology and a remarkable rapidity compared to “omics” analyses.

The systematic screening of patients with cirrhosis by liver ultrasonography every 6 months leads to the disclosure of hepatocellular nodules which do not meet the non-invasive diagnosis criteria for HCC, especially when they are small around 2 cm. A guided biopsy of these nodules has thus to be performed for confirmation of HCC [46]. However, the differential diagnosis between high grade dysplastic nodule and HCC on these biopsy samples remains extremely difficult if not unfeasible and the use of IR microspectroscopy would be greatly helpful in this context.

Hepatocellular nodules disclosed on cirrhotic livers and measuring more than 2 cm are likely to be well differentiated HCC. Four of the 7 premalignant hepatocellular nodules in our series were bigger than 2 cm; only one was reclassified as early HCC and none of the 3 others demonstrated any morphological nor immunohistochemical criteria of HCC. Interestingly, their IR spectral signatures were according with these histological observations.

## Conclusions

This study positions infrared (IR) microspectroscopy as a powerful approach for investigating liver carcinogenesis. The metabolic reprograming that occurs in carcinogenesis constitutes a signature for the diagnosis of precancerous and cancerous lesions. Further studies on larger series of patients will lead to precise the cut off value for discriminating high grade dysplastic lesions and early HCC. Such investigations will have to be performed on paraffin-embedded biopsies that will allow the use of this new method as a routine at the hospital.

## Additional files

10.1186/s12967-016-0763-6 Supplementary Table 1. Assignment of frequency to chemical functions.
10.1186/s12967-016-0763-6 Supplementary Fig. 1 Discrimination between cirrhosis, dysplastic lesions and hepatocellular carcinoma using synchrotron radiation or a laboratory-based infrared source. Spectra were acquired on frozen tissue sections from the patient #37 using SR-FTIR (A, B, E, F, I, J) or using a FTIR microscope equipped with a laboratory-based (Globar) infrared source (C, D, G, H, K, L). Principal component analysis (PCA) was performed on the frequency domain 1480-1360 cm-1. The score plot based on PC1 and PC2 is shown where each point represents one spectrum (A, C, E, G, I, K) and the average spectra corresponding to cirrhotic, dysplastic nodules (DN) or hepatocellular carcinoma (HCC) were extracted and superimposed (B, D, F, H, J, L).

## Abbreviations

CRC: colorectal cancer
DN: dysplastic nodule
FNH: focal nodular hyperplasia
FTIR: Fourier transform infrared
GC: gallbladder cancer
HBV: hepatitis B virus
HCC: hepatocellular carcinoma
HCV: hepatitis C virus
HES: hematoxylin eosin safran
HGDN: high-grade dysplastic nodule
IR: infrared
LGDN: low-grade dysplastic nodule
PCA: principal component analysis
SR: synchrotron radiation
WDHCC: well-differentiated hepatocellular carcinoma
FTIR: Fourier transform infrared
