# Supplementary material for: Discrimination of cirrhotic nodules, dysplastic lesions and hepatocellular carcinoma by their vibrational signature
Source: J Transl Med. 2016 Jan 12;14:9. doi: 10.1186/s12967-016-0763-6 (PMC4710034; doi:10.1186/s12967-016-0763-6)
Supplement: Supplementary file 1 — 10.1186/s12967-016-0763-6 Supplementary Table 1. Assignment of frequency to chemical functions. [file 12967_2016_763_MOESM1_ESM.doc]

**Supplementary Table 1. Assignment of frequency to chemical functions [1,2]**

| **Wavenumber**  **(cm^-1^)** | **Functional**  **group** | **Commonly assigned**  **Biochemical component** |
| --- | --- | --- |
| ~2957 | C-CH_3_ (as) | Lipids |
| ~2920 | -(CH_2_)_n_- (as) |
| ~2872 | C-CH_3_ (s) |
| ~2851 | -(CH_2_)_n_- (s) |
| ~1740 | -CH_2_-COOR | Phospholipid esters |
| ~1655 | O=C-N-H | Amide I peptide, protein |
| ~1545 | O=C-N-H | Amide II peptide, protein |
| ~1450 | -(CH3)n- (as)  -(CH2)n (as) | Lipid, protein |
| ~1395 | -(CH3)n- (as)  -(CH2)n (as)  -O-C=O | Lipid, protein |
| ~1350 – ~1200 | O=C-N-N, CH_3_ | Amide III peptide, protein, collagen |
| ~1250 – ~1230 | RO-PO_2_^-^-OR (as) | DNA, RNA, phospholipid, phosphorylated protein |
| ~1170 | R-COO-R’ (as) | Ester |
| ~1160 and ~1120 |  | RNA ribose |
| ~1150 | C-O, C-O-H | Carbohydrates |
| ~1095, ~1084, ~1070 | RO-PO_2_^-^-OR (s) | DNA, RNA, phospholipid, phosphorylated protein |
| ~1078 | C-C | Glycogen |
| ~1060, ~1050, ~1015 | C-O | DNA and RNA ribose |
| ~1050 | C-O-P | Phosphate ester |
| ~1028 | C-O-H | Glycogen |

as, antisymmetric vibration; s, symmetric vibration.

1 Dreissig I, Machill S, Salzer R, Krafft C. Quantification of brain lipids by FTIR spectroscopy and partial least squares regression. Spectrochim Acta A Mol Biomol Spectrosc 2009; 71: 2069–2075;

2 Banyay M, Sarkar M, Graslund A. A library of IR bands of nucleic acids in solution. Biophys Chem 2003; 104: 477–488.
