# Supplementary material for: Discrimination of cirrhotic nodules, dysplastic lesions and hepatocellular carcinoma by their vibrational signature
Source: J Transl Med. 2016 Jan 12;14:9. doi: 10.1186/s12967-016-0763-6 (PMC4710034; doi:10.1186/s12967-016-0763-6)
Supplement: Supplementary file 2 — 10.1186/s12967-016-0763-6 Supplementary Fig. 1 Discrimination between cirrhosis, dysplastic lesions and hepatocellular carcinoma using synchrotron radiation or a laboratory-based infrared source. Spectra were acquired on frozen tissue sections from the patient #37 using SR-FTIR (A, B, E, F, I, J) or using a FTIR microscope equipped with a laboratory-based (Globar) infrared source (C, D, G, H, K, L). Principal component analysis (PCA) was performed on the frequency domain 1480-1360 cm-1. The score plot based on PC1 and PC2 is shown where each point represents one spectrum (A, C, E, G, I, K) and the average spectra corresponding to cirrhotic, dysplastic nodules (DN) or hepatocellular carcinoma (HCC) were extracted and superimposed (B, D, F, H, J, L). [file 12967_2016_763_MOESM2_ESM.pptx]

## Slide 1
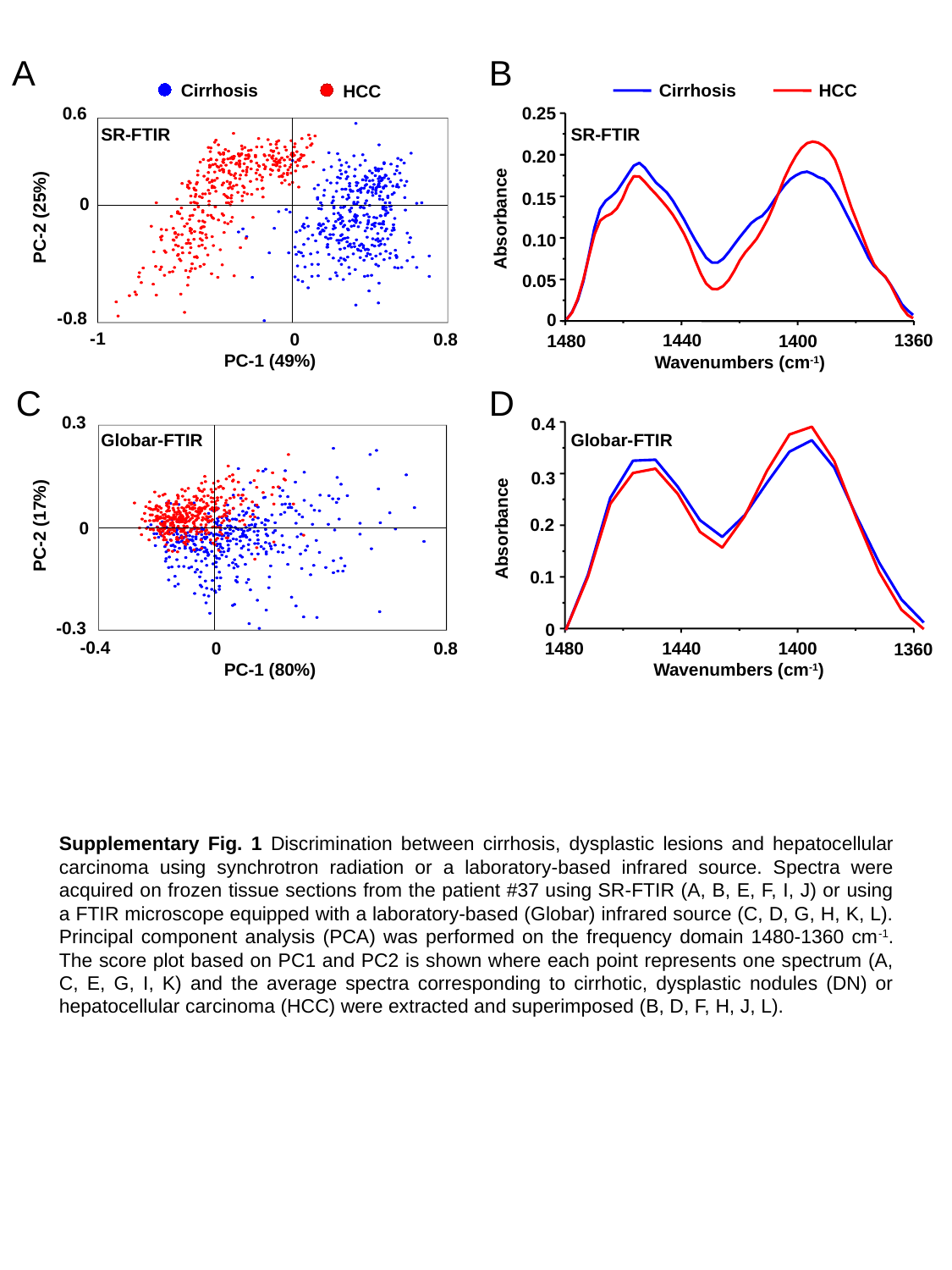

A
B
Cirrhosis
HCC
Cirrhosis
HCC
0.6
0
-0.8
0.25
0.20
0.15
0.10
0.05
0
PC-2 (25%)
Absorbance
SR-FTIR
SR-FTIR
-1
0
0.8
1360
1440
1480
1400
PC-1 (49%)
Wavenumbers (cm-1)
C
D
0.3
0
-0.3
0.4
0.3
0.2
0.1
0
Globar-FTIR
Globar-FTIR
PC-2 (17%)
Absorbance
-0.4
0
0.8
1400
1480
1440
1360
PC-1 (80%)
Wavenumbers (cm-1)
Supplementary Fig. 1 Discrimination between cirrhosis, dysplastic lesions and hepatocellular carcinoma using synchrotron radiation or a laboratory-based infrared source. Spectra were acquired on frozen tissue sections from the patient #37 using SR-FTIR (A, B, E, F, I, J) or using a FTIR microscope equipped with a laboratory-based (Globar) infrared source (C, D, G, H, K, L). Principal component analysis (PCA) was performed on the frequency domain 1480-1360 cm-1. The score plot based on PC1 and PC2 is shown where each point represents one spectrum (A, C, E, G, I, K) and the average spectra corresponding to cirrhotic, dysplastic nodules (DN) or hepatocellular carcinoma (HCC) were extracted and superimposed (B, D, F, H, J, L).

## Slide 2
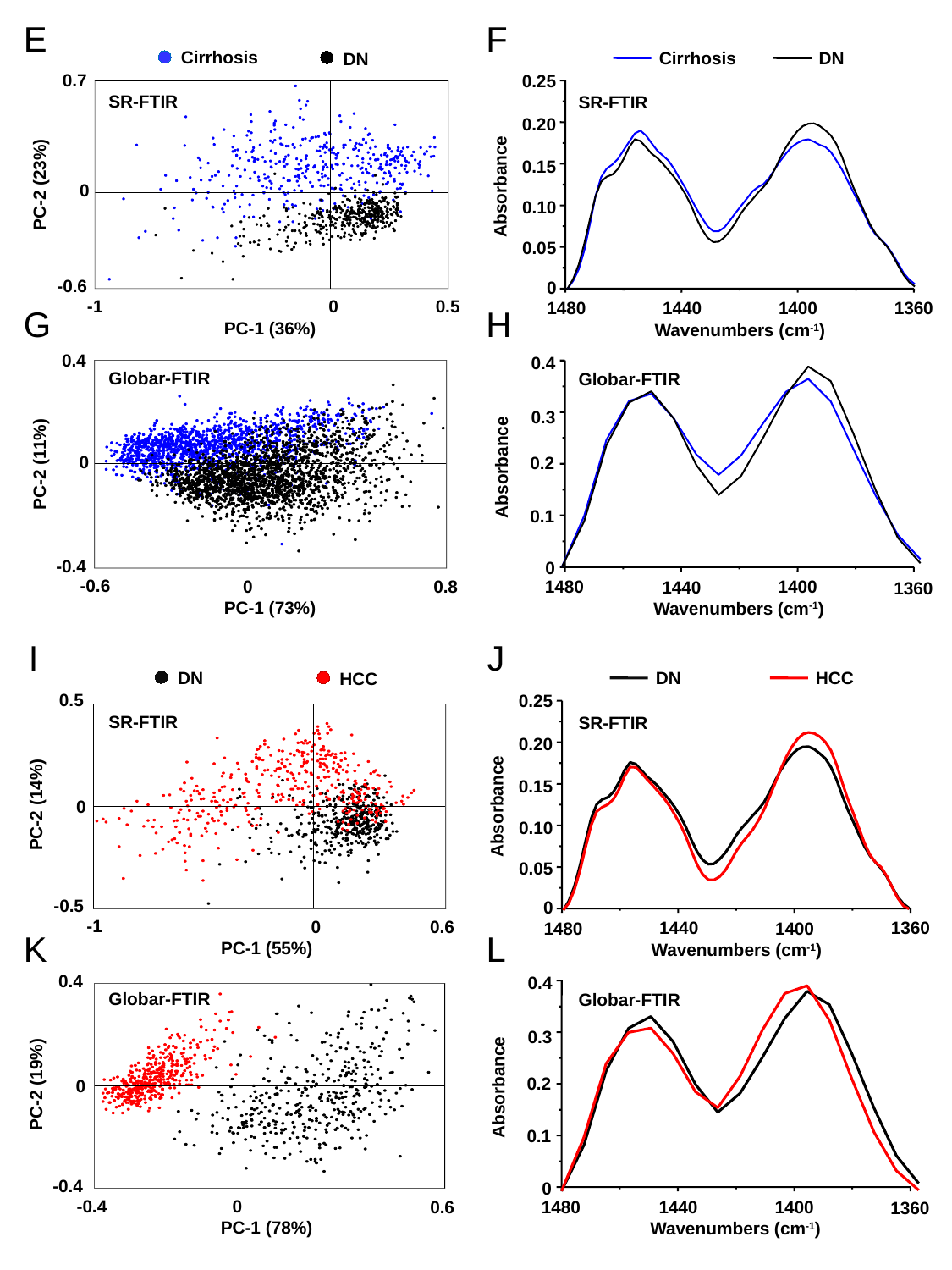

E
F
Cirrhosis
DN
Cirrhosis
DN
0.7
0
-0.6
0.25
0.20
0.15
0.10
0.05
0
PC-2 (23%)
Absorbance
SR-FTIR
SR-FTIR
-1
0
0.5
G
H
1360
1440
1480
1400
PC-1 (36%)
Wavenumbers (cm-1)
0.4
0
-0.4
0.4
0.3
0.2
0.1
0
Globar-FTIR
Globar-FTIR
PC-2 (11%)
Absorbance
-0.6
0
0.8
1400
1480
1440
1360
PC-1 (73%)
Wavenumbers (cm-1)
I
J
DN
HCC
DN
HCC
0.5
0
-0.5
0.25
0.20
0.15
0.10
0.05
0
PC-2 (14%)
Absorbance
SR-FTIR
SR-FTIR
-1
0
0.6
1360
1440
1480
1400
K
L
PC-1 (55%)
Wavenumbers (cm-1)
0.4
0
-0.4
0.4
0.3
0.2
0.1
0
Globar-FTIR
Globar-FTIR
PC-2 (19%)
Absorbance
-0.4
0
0.6
1400
1480
1440
1360
PC-1 (78%)
Wavenumbers (cm-1)
